# Effect of Ethanolic *Caesalpinia sappan* Fraction on In Vitro Antiviral Activity against Porcine Reproductive and Respiratory Syndrome Virus

**DOI:** 10.3390/vetsci8060106

**Published:** 2021-06-09

**Authors:** Chaiwat Arjin, Surat Hongsibsong, Kidsadagon Pringproa, Mintra Seel-audom, Warintorn Ruksiriwanich, Kunrunya Sutan, Sarana Rose Sommano, Korawan Sringarm

**Affiliations:** 1 Department of Animal and Aquatic Sciences, Faculty of Agriculture, Chiang Mai University, Chiang Mai 50200, Thailand; chaiwat_arjin@cmu.ac.th (C.A.); mintra.s@cmu.ac.th (M.S.-a.); 2 School of Health Science Research, Research Institute for Health Sciences, Chiang Mai University, Chiang Mai 50200, Thailand; surat.hongsibsong@cmu.ac.th (S.H.); kunrunya.s@cmu.ac.th (K.S.); 3 Department of Veterinary Bioscience and Veterinary Public Health, Faculty of Veterinary Medicine, Chiang Mai University, Chiang Mai 50100, Thailand; kidsadagon.p@cmu.ac.th; 4 Cluster of Research and Development of Pharmaceutical and Natural Products Innovation for Human or Animal, Chiang Mai University, Chiang Mai 50200, Thailand; warintorn.ruksiri@cmu.ac.th (W.R.); sarana.s@cmu.ac.th (S.R.S.); 5 Department of Pharmaceutical Sciences, Faculty of Pharmacy, Chiang Mai University, Chiang Mai 50200, Thailand; 6 Department of Plant and Soil Sciences, Faculty of Agriculture, Chiang Mai University, Chiang Mai 50200, Thailand

**Keywords:** *Caesalpinia sappan*, porcine reproductive and respiratory syndrome virus (PRRSV), antiviral activity, fractionation, LC–QTOF-MS

## Abstract

Porcine reproductive and respiratory syndrome virus (PRRSV) is a major epidemic in pig production, leading to economic losses in the pig industry worldwide. The use of medicinal plants with antiviral properties might be useful help to prevent and control PRRSV outbreaks. *Caesalpinia sappan* (CS) heartwood is an important herbal ingredient used in Thai folk medicine, possessing various biological activities, including antiviral activity. The present study focuses on the in vitro antiviral activity against PRRSV of a semi-purified fraction of ethanolic CS crude extract using preparative high-performance liquid chromatography. Qualification of the fractions illustrating positive antiviral activity was carried out with liquid chromatography–quadrupole time-of-flight mass spectrometry. The preparative chromatography separated the crude extract into six consecutive fractions, among which the first fraction showed potential antiviral activity by inhibiting PRRSV replication in a MARC-145 monolayer (virus titer 2.75 median tissue culture infective dose (TCID_50_)/mL (log_10_) vs. 9.50 median log10 TCID_50_/mL of the control) at 72 h post-infection, and this fraction included byakangelicin, brazilin, naringenin, and brazilein. These results provide useful information for further study to effectively develop the CS bioactive antiviral compounds against PRRSV as a feed additive or veterinary drug in the pig industry.

## 1. Introduction

Porcine reproductive and respiratory syndrome (PRRS) is an important major infectious disease that has adversely impacted the global pig industry. Porcine reproductive and respiratory syndrome virus (PRRSV), the causative agent of PRRS, is an enveloped single-stranded positive-sense RNA virus that belongs to the *Arteriviridae* (order *Nidovirales*) family [1,2]. PRRSV induces reproductive failure in pregnant sows and respiratory distress in pigs of all ages [3] as well as predisposing pigs to infection by bacteria and other viral pathogens [4,5]. Even though vaccinations have been used to control this disease, some vaccine types, including modified live vaccines, have not been successful in eradicating the virus and do not provide complete immunity from heterologous infections [6,7]. Moreover, there are currently no specific drug treatments in clinical use available against PRRSV infection. The use of antibiotics might help in controlling secondary infections during PRRSV infections [8]. However, the use of antibiotics could promote antibiotic resistance, which has an adverse impact on animal health and is consequently associated with resistant infections in humans [9]. Therefore, attempts have been made to search for medicinal plants with antiviral potential to enable the development of a novel antiviral drug to prevent PRRSV [6,10,11]. Our previous studies discovered that the *Caesalpinia sappan* (CS) crude extract had antiviral activity against PRRSV by inhibiting PRRSV replication in vitro in the MARC-145 cell line [12]. CS is a well-known medicinal plant belonging to the Leguminosae family [13], distributed and cultivated in tropical Asian regions such as Southern China, India, Myanmar, Vietnam, Sri Lanka, and Thailand. The dried heartwood of CS has been used for a long time in oriental folk medicines such as in Indian Ayurveda and Traditional Chinese Medicine [14]. It is also the source of a natural red dye that can be used as a coloring agent in food, beverage, and cosmetics [15]. A decoction made from its heartwood is used in Namya-Uthai as a cardiotonic and to quench thirst [16]. In Northern Thailand, especially in the Chiang Mai, Nan, and Lampang provinces, a CS heartwood decoction is used as an anti-inflammatory agent for the treatment of traumatic disease and arthritis [13]. Many biological properties of CS have been reported such as antioxidant [12,13,17,18], antibacterial [15,16,19], anti-inflammatory [17,20], hypoglycemic [21,22], and hepatoprotective [23] activities. In addition, the constituents of CS extract exhibited high activity against the influenza virus (H3N2) [24]. Bunluepuech and Tewtrakul [25] also reported that CS ethanolic extract could exhibit HIV-1 inhibitory activity. However, defining the exact antiviral agent had been difficult due to the complex phytocomposition of the ethanolic extract. Fractionation is, therefore, very crucial to further characterize bioactive compound groups with antiviral properties. Preparative high-performance liquid chromatography (HPLC) is a classical chromatographic method extensively used in the separation, purification, and upscale of natural products of interest [26]. No data are yet available on the HPLC fractionation of CS extract, which conveys antiviral activity against PRRSV. Therefore, the objectives of this study were separate and collect semi-purified fraction of CS extract by preparative HPLC, to determine each semi-purified fraction with in vitro antiviral activity against PRRSV, and to characterize the responsible bioactive compounds.

## 2. Materials and Methods

### 2.1. Experimental Plan

**Figure 1:**
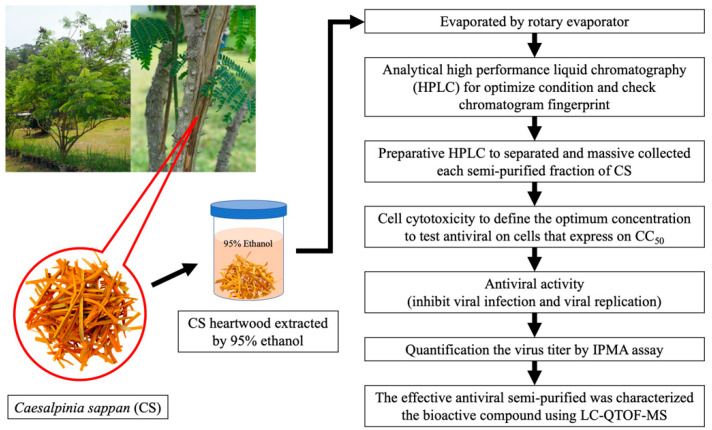
The experimental procedure to determine the CS fraction with in vitro antiviral activity against PRRSV.

This study was designed to identify the CS extract fraction with antiviral activity against PRRSV (Figure 1). A crude extract of CS heartwood was obtained with 95% ethanol. Analytical high-performance liquid chromatography (HPLC) was use to optimize the analysis conditions and chromatogram fingerprint for CS crude extract. Next, preparative HPLC was performed to separate and collect each semi-purified fraction of CS. All semi-purified fractions of CS underwent a cell cytotoxicity test to define the optimum concentration for testing their antiviral activity in cells (expressed as 50% cytotoxic concentration (CC_50_). The antiviral activity for the inhibition of virus infection and replication was determined as a function of the CC_50_. The immunoperoxidase monolayer assay (IPMA) was used to assess virus titration. Lastly, the effective antiviral bioactive compounds in the semi-purified fractions were characterized using liquid chromatography–quadrupole time-of-flight mass spectrometry (LC–QTOF-MS).

### 2.2. Sample Collection and Extraction

The heartwood of CS was collected from Samoeng District, Chiang Mai Province, Thailand. The heartwood was chopped and dried at 60 °C in a hot-air oven for 48 h. Then, it was ground to powder and sieved through a 1 mm mesh. The fine powder (500 g) was macerated with 95% ethanol (3 × 28 L) for 72 h at room temperature, and the extraction was repeated twice. The obtained ethanolic crude extracts were combined and filtered through Whatman No. 1 filter paper. The solvent was completely evaporated using a rotary evaporator at 40 °C. The extracts were stored at −20 °C until further use.

### 2.3. Analytical High-Performance Liquid Chromatography (HPLC)

The ethanolic CS crude extract was dissolved in 10% methanol to a final concentration of 1 mg/mL. It was then semi-purified before identifying fingerprints of the peak using analytical HPLC. The analytical HPLC was performed using an Agilent 1220 Infinity II LC (Agilent Technologies, Santa Clara, CA, USA). Reverse-phase column chromatography was performed using a YMC Triart C-18 (250 × 4.6 mm, 5.5 μm) (YMC, Kyoto, Japan). The mobile phase consisted of 1% acetic acid in distilled water (A) and 1% acetic acid in methanol (B). The gradient elution started at a flow rate of 1 mL/min with 95% A and decreased to 80% A in 5 min, 70% A in 5 min, 65% A in 5 min, 55% A in 5 min, 25% A in 5 min, and 5% A in 5 min before holding for 10 min and returning to 95% A in 5 min. The total run time was 45 min per sample. The photodiode array detection wavelengths were set to 280 and 330 nm.

### 2.4. Semi-Purified Fractionation of CS Extract by Preparative HPLC

The ethanolic CS crude extract was prepared by dissolving it in 10% methanol to a final concentration of 10 mg/mL before passing through 0.45 and 0.22 µm filters. The filtrated CS crude extract was fractionated using a YMC LC-Forte/R recycling preparative HPLC (YMC, Kyoto, Japan) with a photodiode array detector (YMC-YUV-3400). It was operated at 18 mL/min using 1% acetic acid in distilled water (solvent A) and 1% acetic acid in methanol (solvent B). The linear gradient used was as follows: 0 min, 5% B; 5 min, 20% B; 10 min, 30% B; 15 min, 35% B; 20 min, 45% B; 25 min, 75% B; 30 min, 95% B; 35 min, 95% B; and 45 min, 5% B. The preparative HPLC column was a YMC-Actus Triart C18 (250 mm × 20 mm, 5 μm, pore size 12 nm) (YMC, Kyoto, Japan). The detection wavelength was set to 280 and 330 nm. Each injection volume was equivalent to 100 mg/load. Each semi-purified CS extract fraction was collected and evaporated using a rotary evaporator before drying the sample with nitrogen blown through needles onto the samples in tubes. Next, the samples were weighed and kept at −20 °C until they were used for the antiviral bioassay and phytocomposition determination by LC-QTOF-MS.

### 2.5. Cells and Virus

MARC-145 cells (CRL-12231, American Tissue Collection Center (ATCC), Virginia, USA) were grown in Dulbecco’s modified Eagle medium (DMEM) supplemented with 10% fetal bovine serum (Gibco) and 1% penicillin/streptomycin and incubated at 37 °C in 5% CO_2_ in a humidified incubator. PRRSV (VR2332 North American genotype) was propagated in MARC-145 cells, and the virus cells were titrated using IPMA and then stored at −80 °C. Virus titer was determined and expressed as TCID_50_ according to the Reed-Muench method [27].

### 2.6. Cell Cytotoxicity

The MTT (3-(4,5-dimethyl-2-thiazolyl)-2,5-diphenyl-2*H*-tetrazolium bromide) assay was used to determine the effect of the CS extract fractions on MARC-145 cell viability. Briefly, Marc-145 cells were seeded into 96-well plates at a density of 5000 cells/well and incubated in a 5% CO_2_ atmosphere at 37 °C for 24 h. When cells achieved at least 90% confluence, the medium was removed and replaced with a medium containing twofold serial dilutions of the CS extract fraction. The medium without plant extract was used as a control. The plates were incubated at 37 °C under a 5% CO_2_ atmosphere for 72 h. Thereafter, the medium was removed, and 20 μL of freshly prepared 5 mg/mL MTT solution was added to each well, before incubating again at 37 °C for 4 h. Then, the medium was removed and replaced with 150 μL of DMSO to dissolve the crystals, and the plates were shaken for 5 min to dissolve any air bubbles before measuring the MTT signal absorbance at 550 nm. The results were represented as CC_50_, describing the extract concentration that reduced cell viability by 50% when compared to untreated controls [28].

### 2.7. Inhibition of Viral Infection Assay

The inhibition of viral infection assay was performed as previously described [11]. Briefly, the PRRSV at a multiplicity of infection (MOI) of 1 was mixed with media containing CS fractions at the cytotoxicity test concentration and two lower concentrations in twofold dilutions before incubating at 37 °C for 1 h. PRRSV mixed with 1% DMSO served as the positive control. Thereafter, the MARC-145 cells at 5000 cells/well in 96-well plates were inoculated with the mixture of PRRSV and CS fraction or control before incubating at 37 °C for 1 h. Next, the medium was removed and replaced with fresh 2% FBS medium. The supernatant was collected for virus titration 24 h later.

### 2.8. Inhibition of Viral Replication Assay

The inhibition of the viral replication assay was performed as previously described [11]. Briefly, MARC-145 cells were seeded into 96-well plates at a density of 5000 cells/well and infected with PRRSV at an MOI of 1 at 37 °C for 1 h. Then, PRRSV was removed from each well and replaced with media containing diluted CS extract fractions at the concentration from the cytotoxicity test and at two lower concentrations in twofold dilutions. Medium containing 1% DMSO served as the positive control. The plates were cultured under standard conditions (5% CO_2_ atmosphere at 37 °C). The supernatants were collected at 24, 48, and 72 hpi to quantify virus titer.

### 2.9. Virus Titer

The IPMA was used to assess virus titration as previously described [11]. Briefly, cells were fixed with 100 μL of 4% cold formalin for 15 min at room temperature (RT). The fixed cells were washed once with 100 μL of phosphate-buffered saline (PBS) and twice with 100 μL of 0.5% PBS Tween-20 (PBST), before blocking with 100 μL of 1% bovine serum albumin (BSA) in 0.5% PBST for 30 min at RT. Then, the cells were washed. Next, 70 μL of anti-PRRSV NC protein monoclonal antibody (Median Diagnostics, Gangwondo, Korea) was diluted at a ratio of 1:400 and added to cells at RT for 60 min, before incubating again with 50 μL of peroxidase-conjugated AffiniPure goat anti-mouse IgG (H + L) (Jackson ImmunoResearch, West Grove, PA, USA) at a ratio of 1:600 for 60 min at RT. The cells were then washed and counterstained with the 1,5-diaminopentane (DAP) substrate for 5 min before they were washed with distilled water and examined under a microscope. Virus titer was determined using the Reed–Muench method [27], expressed as TCID_50_, describing the virus concentration required to infect 50% of the given cell culture [29].

### 2.10. Characterization of Semi-Purified Fraction by LC-QTOF-MS

LC-MS evaluation was performed using a quadrupole time-of-flight mass spectrometer (QTOF-MS) according to the adopted method of Chellappan et al. [30]. The samples were dissolved in 0.01% formic acid and ethanol (1:1, v/v) to obtain a final concentration of 1 mg/mL and then cleaned up using QuEChERS dispersive SPE kit, fat + pigments (Agilent Technology, Santa Clara, CA, USA) before passing through a 0.22 μm membrane filter. The characterization was performed using an Agilent 1290 Infinity II series, coupled to a 6546 LC/Q-TOF instrument (Agilent Tech., Santa Clara, CA, USA), consisting of a degasser, binary pump, column oven, UV–Vis detector, and thermostat autosampler. The instrument settings were optimized as follows: LC conditions, UV at 330 nm; 0.2 mL/min flow rate; injection volume of 10 μL; and a gradient mobile system starting with 5% ACN and 95% water (1% formic acid), decreasing to 20% ACN in 5 min, 30% ACN in 5 min, 35% ACN in 5 min, 45% ACN in 5 min, 75% ACN in 5 min, and 95% ACN until the run ended. The chromatographic separation was accomplished using a ZORBAX Eclipse Plus C18 (2.1 × 150 mm, 1.8 µm). The MS conditions involved an electrospray ionization (ESI) probe in positive mode. The nebulizer was operated at 20 psi with 7 L/min N_2_ flow. The capillary temperature was kept at 300 °C, while the sample flow rate was set at 8 μL/min. The *m*/*z* range was 50–1000, the capillary voltage was 4500 V, and the dry heater temperature was 280 °C. The chemical structures and other parameters for compounds were determined using an online database (www.chemspider.com, accessed on 1 February 2021).

### 2.11. Statistical Analysis

The cell viability was calculated using a regression analysis of dose–response curves for the MTT assay expressed as CC_50_. Comparisons of the mean of differences in antiviral activities of CS extract fractions were analyzed using a one-way analysis of variance and Tukey’s post hoc test. All statistical analyses were performed using the SPSS 23.0 software (SPSS Inc., Chicago, IL, USA). A *p*-value < 0.05 was considered statistically significant.

## 3. Results

### 3.1. Extraction Yield of Ethanolic CS Heartwood Crude Extract

The ethanolic CS heartwood crude extract yield is shown in Table 1. It was found that a yield as high as 7.020% was achieved from the raw material of CS heartwood (500.323 g) in ethanol.

### 3.2. Analytical HPLC and Preparative HPLC Semi-Purification of CS Extract

**Figure 2:**
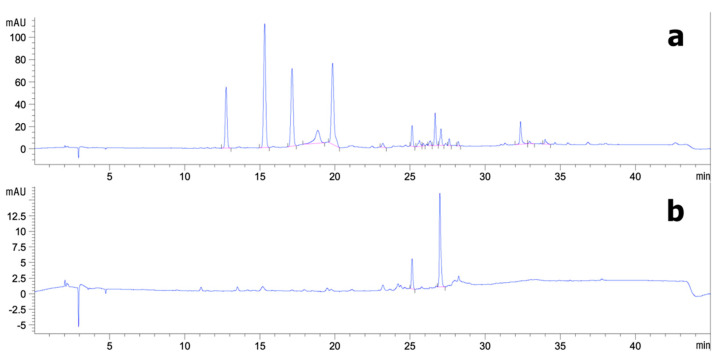
Representative chromatograms of semi-purified CS extract on HPLC UV at (**a**) 280 nm and (**b**) 330 nm.

Figure 2 shows that six semi-purified peaks were marked for preparative HPLC. At 280 nm, the ethanolic CS crude extracts were separated into five dominant semi-purified peaks (A). The yields of semi-purified CS extract fractions are given in Table 2. The CS extract fraction yields ranged from 33.75 to 51.94 mg/g extract. Fraction 1 was isolated with a mobile phase of 30:70 A/B at an Rt of 12.76 min, resulting in 39.54 mg/g extract. Fractions 2–5 were isolated with a mobile phase of 35:65 A/B at Rts of 15.32, 17.14, 18.85, and 19.18 min resulting in 51.94, 42.32, 33.75, and 45.75 mg/g extract, respectively. The results show that F2 was isolated in the highest yield, whereas F4 led to the lowest yield. Moreover, a single dominant peak of 330 nm was apparent at an Rt of 26.99 min (B). Fraction 6 was isolated with a mobile phase of 75:25 A/B, resulting in 37.43 mg/g extract.

### 3.3. Cell Cytotoxicity

**Figure 3:**
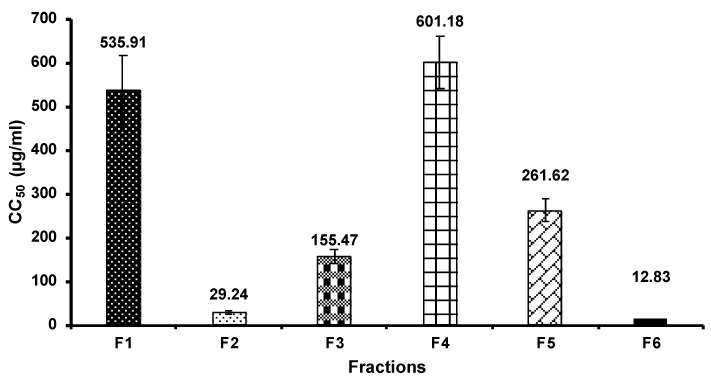
Cytotoxicity of the CS extract fractions toward MARC-145 cells determined by the 3-(4,5-dimethylthiazol-2-yl)-2,5-diphenyl tetrazolium bromide (MTT) assay. MARC-145 cells were incubated with various concentrations of CS extract fractions or control (medium without CS extract fractions) for 72 h prior to the MTT assay. F1, fraction 1; F2, fraction 2; F3, fraction 3; F4, fraction 4; F5, fraction 5; and F6, fraction 6. CC_50_ mean 50% cytotoxic concentration.

Prior to the inhibition of viral replication assay, MARC-145 cell viability was determined. In this study, we expressed the cell viability as 50% cytotoxicity concentration (CC_50_), as shown in Figure 3. The result shows that F4 had the highest CC_50_ of 601.18 µg/mL toward MARC-145 cells, followed by F1 with a CC_50_ of 535.91 µg/mL. On the other hand, we found that F6 had the lowest CC_50_ of 12.83 µg/mL, while F2, F3, and F5 had a CC_50_ of 29.24, 155.47, and 261.62 µg/mL, respectively.

### 3.4. Inhibition of PRRSV Infection

**Figure 4:**
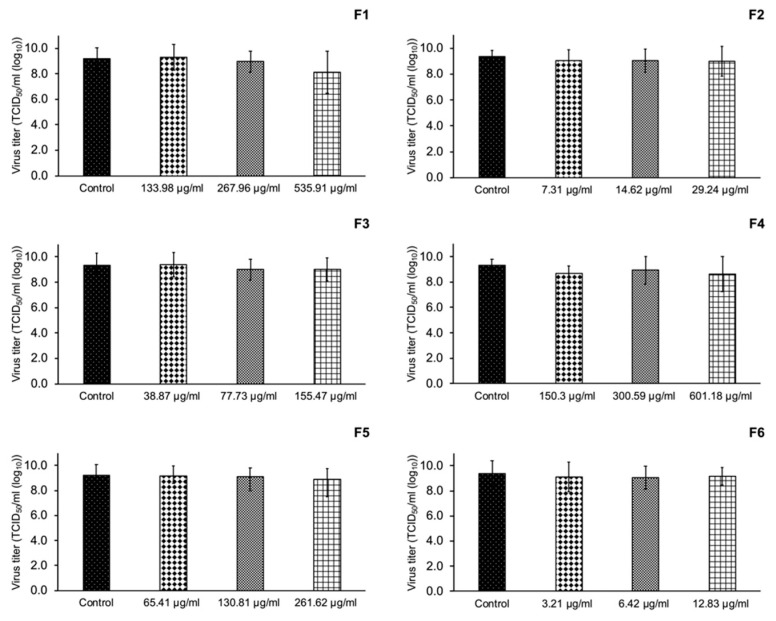
Virus titer describing the inhibition of PRRSV infectivity of six CS extract fractions at 24 h post-infection (hpi). No significant difference for different concentrations of the CS extract fractions. (*p* > 0.05).

Different semi-purified CS fraction concentrations according to the CC_50_ were tested for the inhibition of in vitro viral infection. Our results in Figure 4 show that the semi-purified CS fractions did not significantly inhibit PRRSV infection in MARC-145 cells (*p* > 0.05).

### 3.5. Inhibition of PRRSV Replication

**Figure 5:**
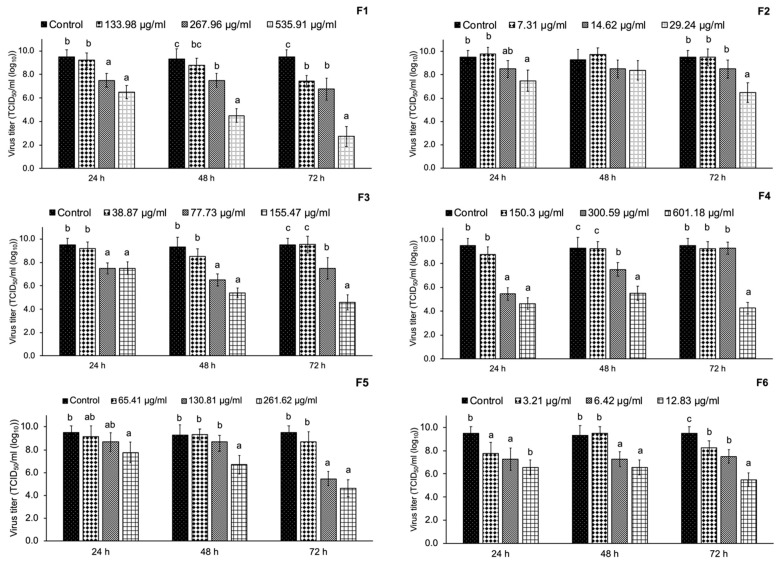
Virus titer describing the inhibition of PRRSV replication of six CS extract fractions at 24, 48, and 72 h post infection (hpi); ^a,b,c^ *p* < 0.05 compared with different concentrations of the CS extract fractions.

**Figure 6:**
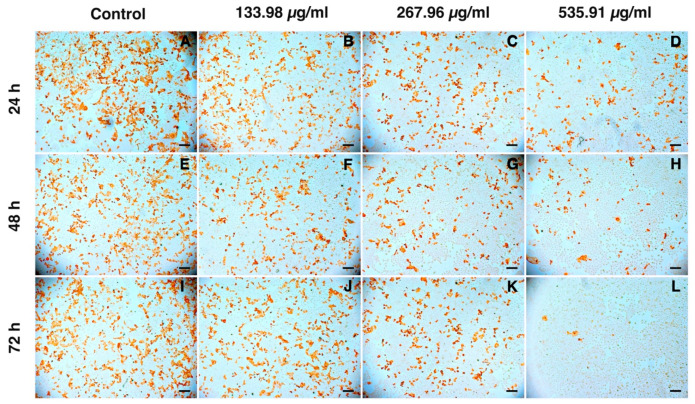
Immunoperoxidase monolayer assay (IPMA) showing the inhibition of PRRSV replication in MARC-145 cells by F1. Scale bar in the figure: 200 μm.

In this study, we treated PRRSV with various concentrations of semi-purified CS extract fractions according to their CC_50_ values as well as two lower concentrations as the range of 12.83–535.91 µg/mL not affecting the proliferative activity of MARC-145 cells at three time intervals (24, 48, and 72 hpi). The results obtained from the antiviral test identified F1 as having significant potential for anti-PRRSV replication activity in a dose-dependent manner (Figure 5). F1 at 535.91 µg/mL showed a stronger inhibitory activity against PRRAV replication at a virus titer of 2.75 TCID_50_/mL (log_10_) at 72 hpi compared with the control (*p* < 0.05). As shown in Figure 6 (brown staining of the cells), the immunoperoxidase monolayer assay (IPMA) indicated that F1 blocked PRRSV replication in MARC-145 cells. Interestingly, F4 at 155.47 µg/mL could inhibit PRRSV replication in MARC-145 cells at a virus titer of 4.25 TCID_50_/mL (log_10_).

### 3.6. Characterization by LC–QTOF-MS

**Figure 7:**
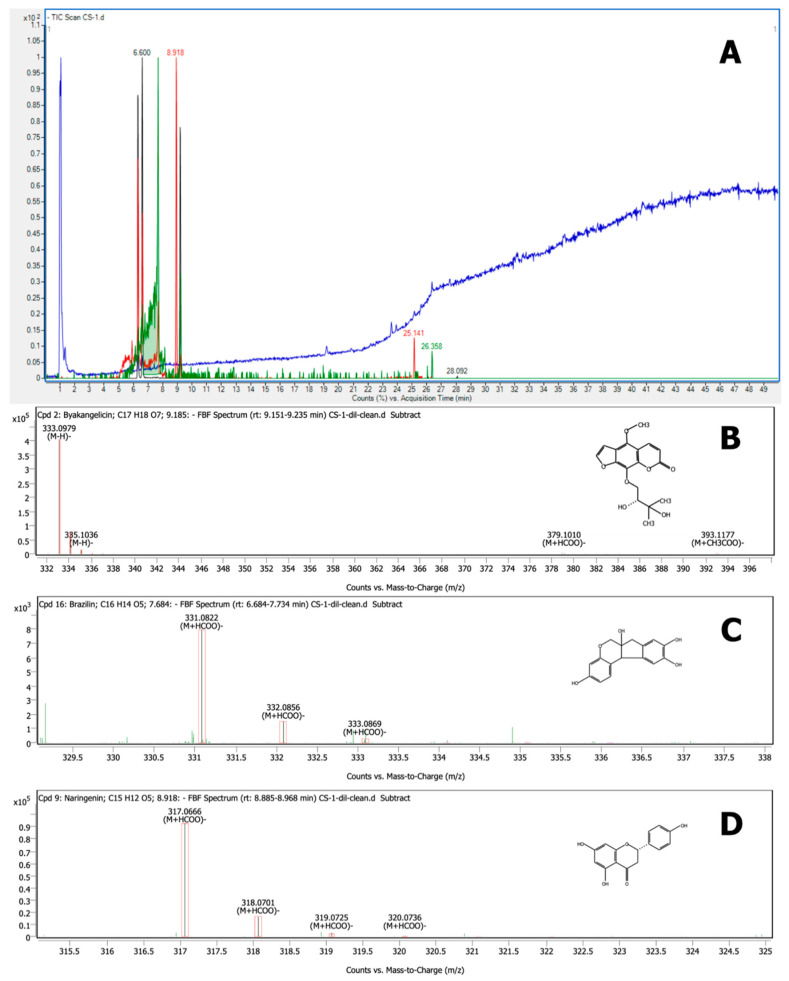
LC–QTOF-MS analysis of F1, along with the top three compounds in terms of matching scores: (**A**) LC–QTOF-MS chromatogram of F1; (**B**) mass spectrum of byakangelicin ((M − H)− = 333.0979); (**C**) single MS spectrum of brazilin ((M + HCOO)− = 331.0822); and (**D**) MS spectrum of naringenin ((M + HCOO)− = 317.0666).

According to its antiviral activity against PRRSV, the most effective fraction was F1. Thus, it was subjected to the identification of bioactive compounds using LC–QTOF-MS analysis. The spectrum showing specific peaks and their corresponding compounds is displayed in Figure 7. The active compounds of F1 revealed from LC–QTOF-MS analysis are listed in Table 3. Fraction 1 had 17 prominent peaks, but only 11 peaks produced a matching score > 90%. Among those, six compounds produced a matching score > 99%, which were identified as byakangelicin, brazilin, naringenin, brazilein, tricin, and wogonin, corresponding to (M − H)– ions at *m*/*z* 333.0979, 285.0768, 271.0612, 283.0613, 329.0665, and 283.0612, respectively.

## 4. Discussion

Medicinal plants represent an alternative source of antiviral agents that can potentially be used instead of antibiotics. The bioactive ingredients of medicinal plants illustrate a wide range of biological properties such as antibacterial, antioxidant, anti-inflammatory, and antiviral activities. As the most commonly identified active compound group, flavonoids are the major class illustrating anti-viral properties [31]. Previous studies have reported that flavonoids, including genistein and catechins, could reduce the infectivity of various viruses affecting humans and animals, including adenovirus, herpes simplex virus (HSV), human immunodeficiency virus (HIV), PRRSV, and rotavirus [32,33]. Many studies also reported the potential of medicinal plants against PRRSV. Kaewprom et al. [34] presented that *Thymus vulgaris* and *Nepeta cataria* hydrosols blocked the viral attachment, adsorption, replication, and release of PRRSV load in MARC-145 cells. Anantikulchai et al. [35] showed that a turmeric extract could inhibit PRRSV replication in vitro. Pringproa et al. [11] found that the crude extract of *Cynodon dactylon* potentially inactivated PRRSV and inhibited replication of PRRSV in vitro. Furthermore, an ethanolic crude CS extract exhibited effective antiviral activity against PRRSV [12]. However, the specific bioactive compound groups in CS crude extract illustrating antiviral properties were not elucidated. Therefore, we separated a semi-purified CS crude extract before identifying the effective compound groups with antiviral activity against PRRSV. Prior to antiviral activity determination, the cell cytotoxicity of fractions was established to avoid cell damage. The fractions with the lowest cell cytotoxicity were F4 and F1 with CC_50_ of 601.18 and 535.91 µg/mL, respectively. Adnan et al. [36] explained that antiviral compounds should be highly effective while showing minimal toxicity to normal cells and tissues.

In this study, the ethanolic CS crude extract was fractioned before testing antiviral activity at concentrations according to the CC_50_. All CS fractions showed no effect against PRRSV in terms of inhibiting viral infection. This is in line with the results previously reported for CS crude extract [12]. Although the CS fractions did not prevent viral infection, they effectively inhibited PRRSV replication, especially F1. F1 was evaluated for its bioactive ingredients by LC–QTOF-MS, revealing that the main bioactive components of F1 were flavonoids such as brazilin, naringenin, brazilein, and catechin. Table 3 lists the compounds with a matching score > 90%. The most dominant compound found in F1 was byakangelicin. Byakangelicin is a major natural coumarin compound found in *Angelica dahurica*, which is a folk medicine in which the roots are used to treat cold and fever in East Asian countries. It also possesses various pharmacological properties, such as antibacterial, antiasthmatic, hypotensive, anti-inflammatory, and antioxidant activities [37]. Likewise, it has been reported that byakangelicin exhibits effective anti-HIV activity [38].

Moreover, other identified flavonoid compounds in F1 were brazilin, naringenin, and brazilein. Brazilin is the main homoisoflavonoid constituent in CS heartwood [39]. Brazilin and brazilein exhibit various pharmacological properties such as anti-inflammatory [40,41], antioxidant [17], and antibacterial activities [42,43]. Laksmiani et al. [44] reported favorable in silico molecular docking studies of brazilin and brazilein from CS heartwood with angiotensin-converting enzyme 2 (ACE2). In addition, they were able to act as entry inhibitors of SARS-Cov-2 by inhibiting ACE2 and transmembrane protease serine 2 (TMPRSS2). Moreover, CS heartwood also inhibited RNA-dependent RNA polymerase (RdRp), which plays a role in RNA replication in the host cell. Nevertheless, until now, no report was available on the antiviral activity of brazilin against PRRSV. We assume that it might play a part in the inhibition of PRRSV replication. To this end, we anticipate that the mechanism of brazilin needs to be investigated in the future in a similar manner to Laksimiani et al. [44]. Naringenin is a flavanone considered one of the most important flavonoids due to its potential biological properties such as antioxidant, anti-inflammatory, and antiviral activities [45,46]. Naringenin is typically found in citrus fruits [45]. Several reports have shown that naringenin exhibits high potential as an antiviral agent. Frabasile et al. [47] found that the administration of naringenin to Huh 7.5 cells could inhibit Dengue virus replication. It is possible that naringenin might inhibit proteases during the virus replication process. Khaerunnisa et al. [48] reported that flavonoids such as naringenin, kaempferol, quercetin, and apigenin are the most recommended compounds that may act as potential inhibitors of the SARS-CoV-2 main protease. Our study, however, classified the complex composition of the CS fraction with the highest potential antiviral activity. Further purification steps of this fraction, accompanied by antiviral activity testing of the purified compounds, may confirm the effectiveness of *C. sappan* heartwood as an anti-PRRSV agent. We report, for the first time, the potential antiviral activity of the detected compounds (including byakangelicin, brazilin, naringenin, and brazilein) toward PRRSV. Future studies can aim to determine the mechanism of action underlying the antiviral activity of these compounds toward PRRSV.

## 5. Conclusions

CS extract has potential anti-PRRSV activity in vitro. The ethanolic CS crude extract was separated into six semi-purified fractions by preparative HPLC. This study showed that only the initial fraction detected at 280 nm illustrated effectiveness against PRRSV in terms of inhibiting viral replication. LC–QTOF-MS analysis revealed the coumarin compound byakangelicin and flavonoids including brazilin, naringenin, and brazilein as the main bioactive compounds in F1, which could play a role as anti-PRRSV agents. These compounds merit further evaluation through purification and structure elucidation.

## Figures and Tables

**Table 1:** The ethanolic crude CS heartwood extraction yield.

Sample	Weight (g)	Yield (g)	% Yield
CS heartwood	500.323	35.124	7.020

**Table 2:** The preparative HPLC fraction yields of ethanolic CS crude extract.

Fractions	Retention Time (min)	Yield (mg/g Extract)
F1	12.76	39.54 ± 5.85
F2	15.32	51.94 ± 5.12
F3	17.14	42.32 ± 5.46
F4	18.85	33.75 ± 4.60
F5	19.84	45.75 ± 3.22
F6	26.99	37.43 ± 4.46

**Table 3:** Compounds identified from F1 of ethanolic crude CS extract according to LC–QTOF-MS.

No.	Compound Name	Structure	Rt	Matching Score (%)	*m*/*z* *	(M − H)−	Mass	Mass Diff (Tgt/ppm)
1	Catechin	C_15_ H_14_ O_6_	4.466	98.52	289.0658	289.0718	290.079	−0.10
2	Isorhamnetin	C_16_ H_12_ O_7_	5.750	95.57	315.0509	315.0510	316.0583	−0.16
3	Kaempferol	C_15_ H_10_ O_6_	5.750	93.02	285.0402	285.0425	286.0476	−0.36
4	(+)–Epicatechin	C_15_ H_14_ O_6_	6.517	97.80	349.0928	289.0718	290.079	−0.13
5	Brazilein	C_16_ H_12_ O_5_	6.600	99.64	283.0613	283.0612	284.0685	0.16
6	Kaempferide	C_16_ H_12_ O_6_	7.051	98.79	299.056	299.0561	300.0633	−0.16
7	Brazilin	C_16_ H_14_ O_5_	7.684	99.83	331.0822	285.0768	286.084	−0.41
8	Naringenin	C_15_ H_12_ O_5_	8.918	99.80	317.0666	271.0612	272.0684	−0.34
9	Byakangelicin	C_17_ H_18_ O_7_	9.185	99.97	333.0979	333.0980	334.1052	−0.27
10	Tricin	C_17_ H_14_ O_7_	9.935	99.10	329.0665	329.0667	330.0738	−0.44
11	Wogonin	C_16_ H_12_ O_5_	9.935	99.10	329.0665	283.0612	284.0683	−0.50

* The *m*/*z* of (+)-epicatechin is shown as M + CH_3_COO^−^ (compound mass + 59.0440), while that of brazilin, naringenin, and wogonin is shown as M + HCOO^−^ (compound mass + 45.0174).

## Data Availability

The data presented in this study are available on request from the corresponding author.
